# Kangaroo mother care diminishes pain from heel lance in very preterm neonates: A crossover trial

**DOI:** 10.1186/1471-2431-8-13

**Published:** 2008-04-24

**Authors:** C Celeste Johnston, Francoise Filion, Marsha Campbell-Yeo, Celine Goulet, Linda Bell, Kathryn McNaughton, Jasmine Byron, Marilyn Aita, G Allen Finley, Claire-Dominique Walker

**Affiliations:** 1 School of Nursing, McGill University, Montreal, Canada; 2 Neonatology, IWK Health Centre, Halifax, Canada; 3 Faculty of Nursing, University of Montreal, Montreal, Canada; 4 School of Nursing, University of Sherbrooke, Sherbrooke, Canada; 5 Department of Anesthesia, Dalhousie University, Halifax, Canada; 6 Department of Psychiatry, McGill University, Montreal, Canada

## Abstract

**Background:**

Skin-to-skin contact, or kangaroo mother care (KMC) has been shown to be efficacious in diminishing pain response to heel lance in full term and moderately preterm neonates. The purpose of this study was to determine if KMC would also be efficacious in very preterm neonates.

**Methods:**

Preterm neonates (n = 61) between 28 0/7 and 31 6/7 weeks gestational age in three Level III NICU's in Canada comprised the sample. A single-blind randomized crossover design was employed. In the experimental condition, the infant was held in KMC for 15 minutes prior to and throughout heel lance procedure. In the control condition, the infant was in prone position swaddled in a blanket in the incubator. The primary outcome was the Premature Infant Pain Profile (PIPP), which is comprised of three facial actions, maximum heart rate, minimum oxygen saturation levels from baseline in 30-second blocks from heel lance. The secondary outcome was time to recover, defined as heart rate return to baseline. Continuous video, heart rate and oxygen saturation monitoring were recorded with event markers during the procedure and were subsequently analyzed. Repeated measures analysis-of-variance was employed to generate results.

**Results:**

PIPP scores at 90 seconds post lance were significantly lower in the KMC condition (8.871 (95%*CI *7.852–9.889) versus 10.677 (95%*CI *9.563–11.792) *p *< .001) and non-significant mean differences ranging from 1.2 to1.8. favoring KMC condition at 30, 60 and 120 seconds. Time to recovery was significantly shorter, by a minute(123 seconds (95%*CI *103–142) versus 193 seconds (95%*CI *158–227). Facial actions were highly significantly lower across all points in time reaching a two-fold difference by 120 seconds post-lance and heart rate was significantly lower across the first 90 seconds in the KMC condition.

**Conclusion:**

Very preterm neonates appear to have endogenous mechanisms elicited through skin-to-skin maternal contact that decrease pain response, but not as powerfully as in older preterm neonates. The shorter recovery time in KMC is clinically important in helping maintain homeostasis.

**Trial Registration:**

(Current Controlled Trials) ISRCTN63551708

## Background

Doing no harm to very preterm neonates is particularly challenging. By virtue of being born too early, before 32 weeks gestational age, the very preterm neonate spends the first several weeks of life in the Neonatal Intensive Care Unit (NICU) where numerous noxious procedures are part of routine care [1-3]. The most common painful procedures are heel lance and intravenous line insertions but topical anesthetics have not been found to be effective in very preterm neonates [4,5]. Sucrose has been repeatedly shown to be effective [6]but frequently repeated doses of sucrose in the very preterm neonate, while effective, may not be safe especially in younger infants [7-9]. Parenteral analgesics either have negative sequellae [10-12] or have not been tested for pain in this population [13]. Behavioral methods of pain control such as non-nutritive sucking, simulated rocking, facilitated tucking, positioning have been tested with non-nutritive sucking having a significant effect, even in very preterm neonates [14-20]. However, reports that mothers find loss of parental role and the pain the infant experiences as being the most stressful aspects of having a child in the intensive care setting [21,22] lead us to explore means of involving mothers to provide comfort during painful events. Breast feeding was found to be effective, but thus far has only been reported to be used for pain control in full-term neonates [23-27]. However, breastfeeding is difficult to establish for very preterm neonates. Results from one study indicate that it may be the contact of breast feeding, as opposed to the breast milk, that is efficacious [28]and this has been supported by results of studies that found breast milk *per se *not to have pain reducing properties [29-32]. Thus for the very preterm group, skin-to-skin maternal contact, or Kangaroo Mother Care (KMC), would appear to be a method which could decrease pain response. Furthermore, it would provide mothers an opportunity to comfort their infant during painful procedures in a technologically invasive environment.

Skin-to-skin contact by the mother, referred to as Kangaroo Mother Care (KMC), has been shown to be efficacious in reducing pain in three previous studies. The first randomized controlled trial was conducted with full-term neonates with results of significant decrease in crying and heart rate acceleration [33]. The first study of KMC in preterm neonates, with restricted age of 32–36 weeks gestational age, had significant decreases in scores of a multidimensional scale that also included behavioral and physiological components [34]. A second study on KMC with preterm neonates included neonates as young as 30 weeks gestational age and it too found decreases in behavioral and physiological outcomes in the KMC group, although whether or not there were differences with the younger group response was not reported [35]. Although there are no more recent published studies on KMC as a comfort measure for procedural pain, there are more studies on KMC and other outcomes [36].

Based on results of animal literature, it had been suggested that infants younger than 32 weeks gestational age may not have the endogenous mechanisms that could be evoked to decrease pain compared to infants above that age[37,38]. Although mechanisms underlying the efficacy of non-nutritive sucking or sweet taste have been debated as endorphin release or some other mechanism such as serotonin release [39-44], it seems clear that some endogenous mechanism triggered by these non-pharmacological strategies is responsible for the analgesic effect in very preterm neonates. This study aimed to test if, like non-nutritive sucking [19] and sucrose [6], kangaroo maternal care could also be effective in decreasing pain response to routine heel lance in infants less than 32 weeks gestational age.

## Methods

### Recruitment

The protocol and consent forms were reviewed by the constituted institutional research ethics review board of each participating centre, namely, the Montreal Children's Hospital, the IWK Health Centre, and Hôpital Ste. Justine. These committees approved the incubator-control condition without sucrose following discussion with staff of the participating units where sucrose was not considered standard care in younger preterm neonates due to perceived safety concerns. The study took place in three level III units, all of which admitted both inborn infants as well as transfers. All supported KMC but did not systematically promote it and none had standard of care policies at the time of the study. Ventilated infants were rarely allowed into KMC and staff comfort with smaller infants varied.

Mothers and their preterm neonates were eligible for participation in the study if the infants met the following criteria: were born between 28 0/7 and 31 6/7 completed weeks post menstrual age (pma) determined by ultrasound at 16 weeks, had informed parental consent, had Apgar scores >6 at 5 minutes, were within 10 days of birth, were breathing unassisted, did not have any major congenital anomalies, had not suffered Grade III or IV intra-ventricular hemorrhage or subsequent peri-ventricular leukomalacia, had not undergone surgery, and were not receiving paralytic, analgesic, or sedative medications within 48 hours. Mothers had to be willing and able to hold their infant in the KMC position for the study. The protocol was explained to the mother who was told about the two conditions lasting 15 minutes of undisturbed time in order for the infants to be in a true baseline state. For practical purposes, if the infant was to be discharged before needing two sessions of blood work, mothers were not approached to participate in the study. Using data from an earlier maternal kangaroo care study [34] using our primary outcome with a mean difference of two points and a standard deviation of 4.5 points, sample size for a power of 0.9 and significance level set to .05, was 55. (PowerSample Size) [45].

### Procedure

Employing a *single-blind crossover design *each infant was to undergo heel lancing for blood procurement for clinical purposes in either KMC position or usual incubator situation within 4 days of each other. Due to the infrequency of blood sampling which was determined by clinical considerations, we allowed a wider window of post-natal age such that there was a minimum of 24 hours and a maximum of 14 days between conditions. Ordering of conditions was determined randomly by a computer-generated program in the study centre and assignment was accessed on the website by the site research nurse after consent was obtained. In the KMC condition, the diaper-clad infant was held upright, at an angle of approximately 60°, between the mother's breasts, providing maximal skin-to-skin contact between baby and mother. A blanket and then the mother's clothing were placed over the infant's back and tucked under each side of the mother. The baby remained in this condition at least 15 minutes prior to heel lancing procedure. Fifteen minutes is shorter than in our earlier study with older preterm neonates, and this time was determined according to acceptance by the staff for whom KMC was not routine. We had also noted that physiological stability and deep sleep typically occur within a minute of being placed in KMC. We asked that the mother keep her hands clasped behind the infants' back throughout the procedure and refrain from touching the infant's head with her face (to keep observers blind). The mother was allowed to speak to her infant since there was no audio recording during the procedure. In the control condition, the baby was placed in the incubator in a prone position, swaddled with a blanket (with heel accessible), for at least 15 minutes prior to the heel lancing procedure. Prone position was selected since it controlled for the frontal pressure component of KMC, allowing us to test the maternal proximity component, as well as the fact that it is recommended for preterm neonates [46,47].

The heel lancing procedure includes five phases. One minute of baseline was collected at the end of the 15 minutes in the assigned condition, that is following 15 minutes of KMC or in incubator. The heel warming phase lasted 1 minute. The heel was then swabbed and lanced with a spring loaded lancet (Tenderfoot ^®^). The instant of lancing was the point at which changes from baseline was determined and was analyzed in 30 second blocks from that instant. An adhesive bandage was applied to the site immediately after all blood was procured. This was the point that indicated the end of the blood sampling procedure. Return to baseline was calculated as time from adhesive bandage application until baseline HR was achieved. There was continuous video, but not audio, recording and pulse oximeter monitoring the heart rate and transcutaneous oxygen saturation of the infant throughout the session, both of which always occurred in the morning after the infant was fed. The continuous data were analyzed in allocated blocks of time and averaged for each phase of the procedure.

### Measures

The primary outcome was the *Premature Infant Pain Profile (PIPP*)[48,49]. The PIPP is a composite measure of pain including physiological (heart rate, trancutaneous oxygen saturation), and behavioral (facial action) indicators and includes weights for younger gestational age and sleep state. Physiological scores are calculated based on changes in maximum heart rate and minimum oxygen saturation changes from baseline. The scores are totaled so that with the seven components scores can range from 0–21, and a difference of two points between conditions can be considered clinically important. The PIPP has been tested for reliability, construct validity and clinical utility, all with results indicating excellent psychometrics [49-51]. One of the strengths of the PIPP is that it accounts for infant contextual variables known to influence pain response, specifically behavioral state at baseline and gestational age. Since in earlier studies KMC put almost all infants into quiet sleep this poses a problem. According to PIPP protocol, we measured baseline state after the infant had been in the condition. There are additional pain score points if an infant is in quiet sleep, which would decrease any differences between conditions if KMC indeed put infants into quiet state, that is, there are additional pain score points given if infant is in quiet sleep during baseline. A second problem in using the PIPP in this study is that all the infants were in the same age range, so there would be no variance in the age factor of the PIPP. Therefore we also analyzed the individual components of the PIPP, ie facial actions, heart rate, and oxygen saturation, in order to compensate for the background factors of behavioral state and gestational age.

*Heart rate *was collected using four ECG leads connected to a data acquisition system (Compumedics E-series) with a sampling rate of 100 Hz averaged on a beat-to-beat basis. *Transcutaneous oxygen *saturation was collected via infrared oximeter (Massimo RadicalO placed on a hand or the unaffected foot of the infant and connected to the data acquisition system. The physiological data were analyzed using the software in the system (Compumedics E-series Profusion PSG II) that allowed minimum, maximum, mean, and standard deviation to be calculated. Artifacts were removed according to protocol in our laboratory which deleted sections in which HR was below range for 4 or more consecutive beats before analyzing. The *three facial actions *(brow bulge, eye squeeze and naso-labial furrow) of the PIPP were continuously recorded by a digital video camera Panasonic KS162 that allows for close range, high quality facial images. This camera was wired into the physiological data acquisition system, with the research nurse striking keys on the computer and flashing color coded cards into the camera to mark phases of heel lancing procedure. Since both physiological and video data were fed into the same data acquisition system, the time stamps were synchronous. The camera was in close up focus on the infant's face with very little surrounding area, no sound, with minimal color, and turned to an angle in the kangaroo condition as to mimic the prone position in order to decrease possibility of unblinding by research assistants who scored the tapes. Research assistants, who were blinded to the purpose of the study by being told that the study was about infant facial actions, coded facial actions in the laboratory of the PI (CJ). The three facial actions were scored according to the Neonatal Facial Coding System [52-55] that provides a detailed, anatomically based, and objective description of newborns' reactions to the heel lance. The selected facial actions were scored on a second-to-second basis. The video-recordings were viewed in real time on Windows Media Player which allowed viewing of the Panasonic AG-1970 default screen with clock to the 4^th ^decimal place. Each recording session was scored three times, once for each of the facial actions, using a laptop computer with software developed in the lab based on BASIC software that records the scores and allows for information on artifacts to be included. A final score based on percentage of time the facial action was present was calculated each 30 second time block throughout each phase of the procedure. The neurobehavioral state component was determined according to Prechtl's categories of quiet sleep or quiet awake or active sleep or active awake [56,57]during the baseline. Gestational age was taken from the chart, based on ultrasound at 16 weeks.

*Severity of illness*, as a potentially confounding variable, was scored using the *Score for Neonatal Acute Physiology Version II (SNAP-II *[58]) for the 12-hour period after birth and in the 12 hour period prior to each study session. The elements for this score can be found in the medical record and include hemodynamic, respiratory, hematological, metabolic, electrolytic, and neurologic parameters. The score has predictive validity for perinatal mortality.

## Results

**Figure 1:**
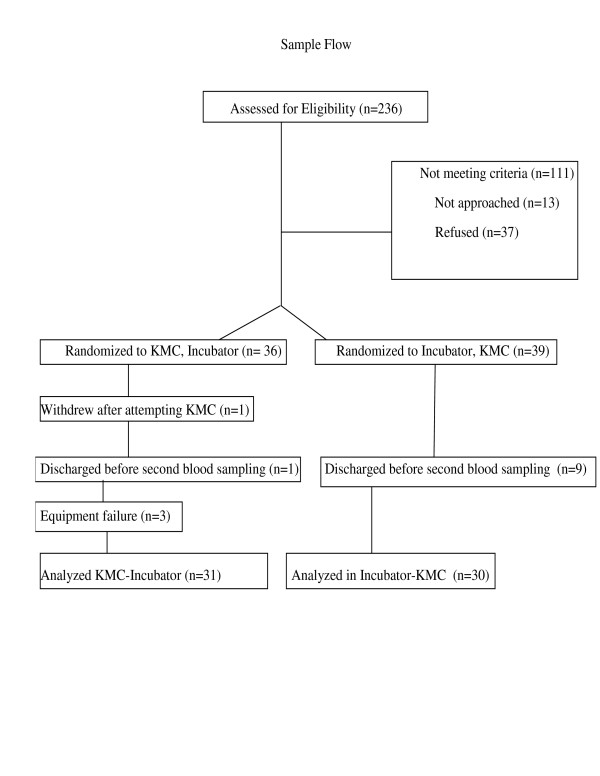
Sample Accrual and Flow.

Across the three sites, there were 236 infants admitted during the data collection period (April 2003-December 2005). (Figure 1) Of those 125 meeting the selection criteria, 114 were approached, and 77 accepted to participate, giving a refusal rate of 32 percent. The main reasons for refusal were that mothers: felt too stressed to participate, did not want anything extra done to their infant, and did not want to see the baby in pain. One mother withdrew when attempting KMC for the study because she felt "too nervous". Physiological and behavioral data were completed for both KMC and control sessions on 64 infants, however data were incomplete (face obscured, EKC lead detaching) on three. The primary reason that infants were lost to the study was that the infant was discharged from the unit or did not require bloodwork within the time frame of the study. The 61 infants remaining in the study were a mean age of 30.5 weeks (SD 7 days), at birth weighed 1421 gm (SD 490 gm), had 5-minute Apgar scores of 8.2 (SD 1.3), and SNAP-PE-II score of 10.08 (SD 10.9). There were significant differences in weight and age between the two sessions (Table 1). Order of condition, postnatal age, or weight had no effect on the pain response. Since gestational age and SNAP scores were not correlated with any outcomes (*r *< .15) they were not included in the analyses as covariates. Means for outcomes between sites and order of condition were almost identical and thus neither of these factors were included in analyses. A repeated measures analysis of variance with condition (KMC vs. incubator) as the repeated factor was conducted for each 30 second period following heel lance through 2 minutes when the majority (83%) of the heel lance procedures had been completed. Thirty-one infants underwent KMC before the incubator condition. Although the blood sampling procedure was17 seconds shorter in KMC than incubator (153 vs 170 seconds), this was not significant. Average baseline heart rate was within 1 beat per minute and oxygen saturation levels within 0.2% between conditions. Behavioral state was different at baseline with 60% of infants in quiet sleep in KMC condition versus 30% in incubator condition (Χ^2 ^(3) = 50.9, *p *< .0001).

**Table 1:** Sample characteristics at each session

	Mean age in postnatal days (sd)*	Mean weight in grams (sd)***	**Mean SNAP-II scores (sd)**
**Session 1 (31 KMC, 30 Incubator)**	220 (6.57)	1362 (267)	3.06 (7.03)
**Session 2 (30 KMC, 31 incubator)**	223 (7.16)	1438 (276)	3.21 (7.06)

*p < .05 ; *** *p *< .001

**Figure 2:**
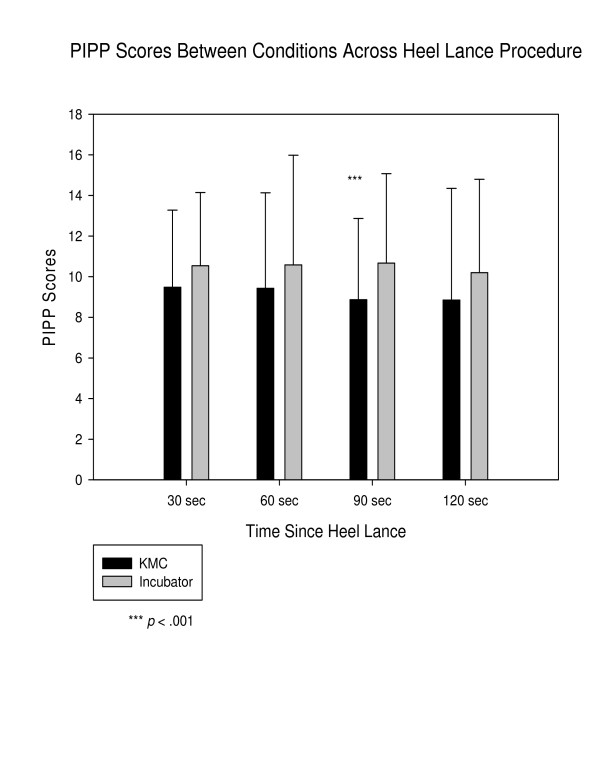
Pain on Premature Infant Pain Profile (PIPP) scores by condition.

Mean pain scores (PIPP) (see Figure 2) were not significantly lower, in the KMC condition at 30 and 60 seconds post-heel lance. By 90 seconds post-heel lance, the difference between scores by condition was significant (KMC 8.871 (95%*CI *7.852–9.889) versus Incubator 10.677 (95%*CI *9.563–11.792) *p *< .001). The difference continued to 120 seconds, although fell short of significance (8.855 (95%*CI *7.447–10.262) versus 10.210 (95%*CI *9.030–11.389) *p *= .145). The time to return to baseline heart rate following the application of the adhesive bandage signifying the end of blood sampling was significantly different, 123 seconds (95%*CI *103–142) for the KMC condition and 193 seconds for incubator condition (95% *CI *158–227) (*F *(61,1) = 13.6, *p *< .0000).

**Figure 3:**
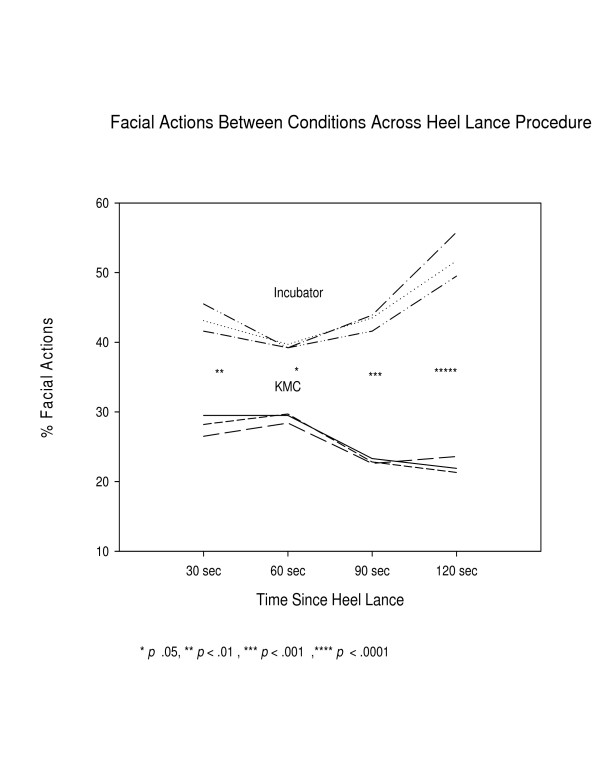
Facial Actions by condition following heel lance.

**Figure 4:**
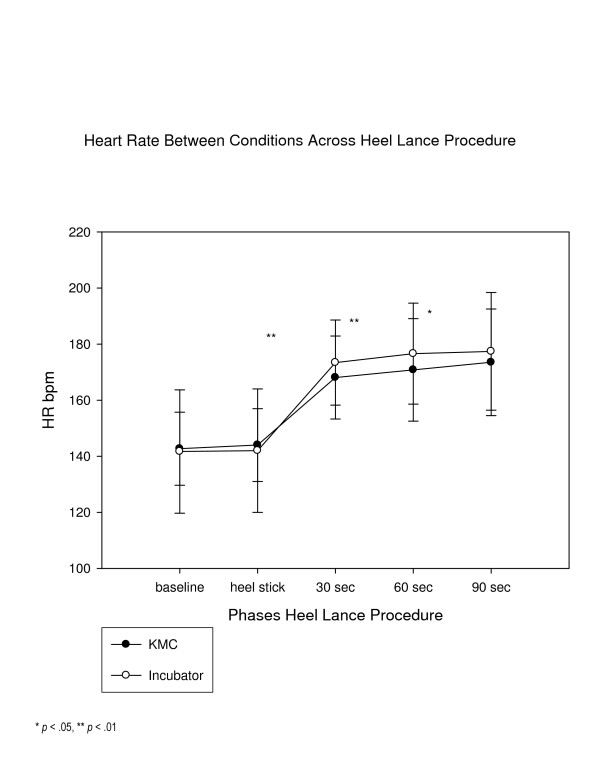
Mean heart rate by condition.

**Figure 5:**
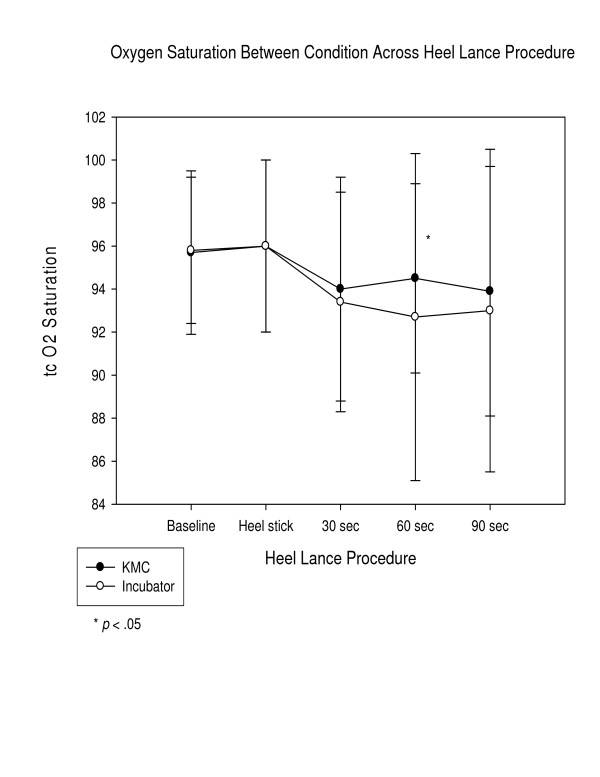
Mean trancutaneous oxygen saturation by condition.

In examining the average physiological indicators and facial actions of the PIPP, facial actions were significantly lower in the KMC condition than the incubator condition (See Figure 3) throughout the phases, reaching a two-fold difference by 120 seconds, and average heart rate was significantly lower at 30, 60, and 90 seconds post-heel lance and (See Figure 4). Average oxygen saturation levels were significantly higher at 60 and 90 seconds post-heel lance (See Figure 5). The physiological differences were calculated on average value, dissimilar to how calculated in the PIPP.

## Discussion

Maternal contact in the skin-to-skin paradigm of KMC decreases pain response in preterm neonates between 28–32 weeks gestational age who are undergoing a heel lance for blood procurement, although the magnitude of the difference is less than 2 points on the 21-point outcome measure, found in our report of infants 32–36 weeks [34]. The differences between incubator and KMC were approximately between 1.1 and 1.8 in the first three 30 second blocks of time, out of a total possible score of 21. While the levels reached statistical significance for some of the phases, and the mean individual components of the PIPP reached statistical differences, the magnitude of the effect was smaller than estimated, based on our earlier study of 32–36 weeks gestational age infants [34]. The effect of KMC was not immediate following the heel lance, as in the study with the older preterm neonates, but was evident further into the heel lance procedure, not until 90 seconds post lance. This delay in effect after the lance was curious since more infants were in quiet sleep during baseline in the KMC condition, and quiet sleep dampens pain response [59]. It appears then, that while preterm neonates less than 32 weeks gestational age do have some endogenous mechanisms that can be invoked through maternal skin-to-skin contact, its effect is not as powerful and it is not as quickly activated as in older preterm neonates.

The issue of when to take baseline measures for the PIPP when the intervention begins many minutes before the heel lance procedure needs addressing. According to PIPP guidelines, baseline measures of state, heart rate and oxygen saturation levels are recorded just prior to the actual procedure, such as the heel lance. In studies such as this when the intervention occurs before the baseline measures would normally be recorded, the values of state, heart rate and oxygen saturation levels are not at baseline levels, because KMC has a modifying effect on each of these parameters. Future research with KMC should take baseline measures before putting the infant into KMC to reflect true baseline measures.

Perhaps more importantly, was the significantly quicker time to recovery. Of clinical interest on procedural pain in very preterm neonates are response, that is the degree to which they respond, and recovery, how quickly they return to pre-procedure state. The ability to recover quickly is a sign of ability to maintain homeostasis, a major task that the very preterm neonate must accomplish in order to grow and develop [60-63]. Facilitation of homeostasis maintenance through KMC has been reported regarding temperature, state, oxygen saturation levels, and growth [62-70] but not in the context of the additional stress of pain. The results of this study indicate that maternal contact can facilitate not only a diminished response, but a quicker recovery in infants between 28 and 32 weeks gestational age.

There are some explanations other than maternal contact for the results. It was impossible to blind the person conducting the heel lance procedure, so that they may have been gentler during that condition. Anecdotally however, they preferred the incubator condition since conducting the procedure in KMC meant the person procuring the blood sample had to bend over towards the infant or be seated on a stool next to mother and infant, not standing next to incubator. Additionally the mother would be observing and some staff were not comfortable with that. When the infant was in KMC, gravity may have helped the blood flow and made the procurement faster, although the 17 second difference was not significant.

Infants in this study were not intubated or even requiring supplemental oxygen, according to the protocols of the units at the time the study began. Now, some intubated infants are permitted to be in KMC and it would be interesting to see if KMC is efficacious for procedural pain in a similar age group, but intubated population. One study on KMC in neonates less than 28 weeks showed that those infants[71] could not maintain temperature in KMC, and until other studies contradict that, studying KMC for pain control in infants less than 28 weeks may not be indicated at this time.

Kangaroo Mother Care for pain management in preterm neonates is obviously cost-effective and has now been shown to be effective in infants from 28 weeks through term. Mothers should be offered KMC as NICU policy, not only to be close to their infant, but also to provide comfort. It is not known if KMC is commonly included as a non-pharmacologic intervention for procedural pain in NICU's but based on results here as well as earlier studies with older preterm neonates, it would be recommended, alone or in conjunction with other strategies such as sweet solutions[6].

## Conclusion

Very preterm neonates between 28–32 weeks gestational age can benefit from KMC to decrease pain from heel lance procedures.

## Competing interests

The authors declare that they have no competing interests.

## Authors' contributions

CJ was responsible for all stages of the study. FF, MCY, LB participated in the protocol development, oversaw data collection from one site, participated in data analysis and manuscript preparation and review, CG, GAF, CDW participated in study design, interpretation of findings and manuscript preparation, MA participated in developing physiological data acquisition protocol, analysis and interpretation of physiological data, manuscript preparation and KM, JB trained and oversaw data coding, data entry and verification, literature review update, and manuscript preparation.

## Pre-publication history

The pre-publication history for this paper can be accessed here:


